# Views, Attitudes and Challenges When Supporting a Family Member in Their Decision to Travel to Switzerland to Receive Aid-In-Dying

**DOI:** 10.3389/ijph.2024.1607410

**Published:** 2024-06-24

**Authors:** Daniel Sperling

**Affiliations:** Department of Nursing, University of Haifa, Haifa, Israel

**Keywords:** suicide tourism, assisted suicide, aid-in-dying, family members, grief and bereavement, Dignitas, Israel

## Abstract

**Objectives:**

Exploring the experiences, perceptions and meanings of family members and close friends of Israeli individuals who sought aid-in-dying outside Israel.

**Methods:**

Using the phenomenological-interpretive approach, a qualitative research design was employed, based on ten in-depth semi-structured interviews with Israelis who had provided support for a relative who embarked on suicide tourism.

**Results:**

The following five themes emerged from interviews: (1) facilitators for supporting an individual requesting suicide tourism; (2) choosing death and actively making the decision to die; (3) the meaning of traveling to die; (4) offering support throughout the process; and (5) facilitating procedures after death.

**Conclusion:**

The participants spoke of the active role that they played in their relative’s suicide-tourism journey. They conveyed conflicting emotions and values regarding the decision at hand, the ability to say goodbye thanks to their pre-planned death, helping to reduce their suffering and burden, and dealing with the challenge of disclosing the deceased’s plans, before and after the act, as well as their own involvement in the process. Relatives of suicide-tourism patients should receive professional support during and following this difficult process.

## Introduction

Aid-in-dying, also known as assisted dying or assisted suicide [1], is the practice whereby individuals receive assistance, usually from physicians, to end their lives due to sickness and suffering [2]. Despite ethical challenges [3–5], the practice, support, and legislation regarding aid-in-dying have increased worldwide over the past decades [1, 6]. With more countries legalizing such practices [7], public interest and discourse has also increased [8].

Advanced life-saving technologies and life-prolonging procedures have also increased [9], yet these may come at a cost, possibly extending the patients’ suffering and diminishing their quality-of-life. In turn, this may enhance the individual’s motivation to end their life unnaturally. However, in countries where aid-in-dying is prohibited, doing so may prove challenging. One possible solution is suicide tourism, where the patients travel to another country where aid-in-dying is legal. Yet despite the increasing popularity of suicide tourism [10–13], the phenomenon still evokes criticism among certain policymakers [14], end-of-life activists [15], and members of the public [13].

### Aid-In-Dying and Suicide Tourism

Studies explore the perspectives of patients, caregivers, right-to-die professionals, and the public regarding aid-in-dying, addressing issues such as poor quality-of-life, dependency, loss of self, and the ability to exercise choice [16–18]. Research also presents the views of individuals seeking aid-in-dying, who express their distress, uncertainty about their future, and fear of prolonged suffering [17, 19–21].

Media coverage and interviews with individuals who are considering suicide tourism convey this option as a “last resort,” yet one that restores meaning to the suffering individual and supported by the aesthetics-of-a-good-death concept [22–24]. Research also shows that many had already experienced suicidal thoughts and/or attempts, indicating that they do not necessarily need their loved ones beside them when they die, or care about which country they die in [23].

### The Role of Family in Aid-In-Dying

Investigating the role of family members who support the aid-in-dying of their loved ones is greatly important, especially regarding suicide tourism that entails travelling to a different country. In addition to the impact that this practice has on family members [25], individuals who are contemplating life-and-death decisions often take into consideration the views of their loved ones [26, 27]; in some cases, placing even greater emphasis on the latter’s opinion than on their own desires [28].

Yet research is lacking on the perspectives of family members who are involved in aid-in-dying decisions and practices [29]. Their involvement could be invaluable to the individual, helping them say goodbye to friends and family and filling a range of supportive roles [25]. According to [30], family involvement takes place in five stages: (1) contemplation of assisted suicide; (2) gaining acceptance and patient-family cooperation for creating the individual’s end-of-life plan; (3) gaining permission, assuring all requirements have been met; (4) organizing the assisted suicide; and (5) dealing with the aftermath, including the family’s feelings following the death of their loved one, while disclosing that assisted suicide took place.

Studies further demonstrate the family-members' ambivalence regarding the individual’s choice of assisted suicide, while showing their respect for the patient’s autonomy and desired relief from suffering [31]. In light of the Because guilt, secrecy and stigma related to assisted suicide, families usually experience more challenging bereavement, as they come to terms with the individual’s choice, seeking comfort in knowing that their loved one is no longer suffering [31–34]. Bereavement complexity may also be heightened by family conflicts about legal aspects, logistical challenges, and negative outside reactions [35].

To the best of the author’s knowledge, only one study (a doctoral dissertation) has examined the experiences of family members who supported an individual’s plan to travel for aid-in-dying, mainly from the UK to Switzerland or Belgium [36]. This study focuses on the family’s bereavement, while emphasizing the unique challenges that are entailed in suicide tourism. For example, conflicts between family members in light of the individual’s wish to travel abroad for aid-in-dying, concerns about a possible legal penalty upon returning home, the painful experience of accompanying their loved one, and difficulty in informing others about the death and related circumstances.

### Aid-In-Dying in Israel

In Israel, end-of-life care is regulated by the 2005 Dying Patient Act, which reflects the compromise between religious, ethical and professional key players, and combines both liberal values and the “sanctity of life” principle [37]. Such a unique approach is reflected in allowing the withholding of life-sustaining treatment at end-of-life under specific circumstances, legally enforcing advance directives and acknowledging a universal right to access palliative care services on the one hand, but prohibiting aid-in-dying and euthanasia, on the other hand.

As such, some Israelis seek aid-in-dying in Switzerland. At the end of 2022, 121 Israeli members were registered with Dignitas, and an increase was seen in the number of accompanied suicides of Israelis, from seven cases in 2018 to 24 cases in 2022 [38]. To add to the literature on suicide tourism, the aim of this unique study was to explore the experiences and attitudes of Israelis who supported a family member or close friend in their aid-in-dying journey outside Israel.

## Methods

### Research Design

The study applied a descriptive qualitative research design, based on the phenomenological-interpretive approach, as a means for exploring the participants’ experiences and interpretations [39].

### Research Population, Sample, and Sampling

The research population included adults from Israel who had provided support to a family member or close friend before or during their aid-in-dying journey to Switzerland. Using purposive sampling, the participants were recruited through a search of media coverage regarding cases of suicide tourism from Israel during 2019–2022, and providing that the participants were able to elucidate the research phenomenon [40]. The researcher contacted 14 potential participants. Following an initial telephone conversation, four decided not to participate in the study, due to lack of interest or concerns about the possible emotional burden that it could place on them. Participants were recruited until thematic saturation was achieved, resulting in 10 participants.

### Data Analysis

Data were collected through in-depth semi-structured interviews, conducted in Hebrew by the author of this article, each lasting 40–170 min. With the participants’ consent, the interviews were recorded and transcribed. They were conducted at a time and place of the participants’ choosing, mainly in their homes, except for one interview that was conducted via Zoom since the participant no longer lived in Israel. An interview guide was developed for this research (Annex S1).

Based on the interpretative phenomenological analysis, the interviews were analyzed in four stages [1]: the transcripts were openly read and re-read a number of times, to allow annotation of interesting or significant things [2]; preliminary annotations were transformed into concise phrases and temporary codes, which were then gradually transformed into themes and re-organized into a scheme of codes and central themes [3]; categories/sub-themes were generated through a process of continuous, fluid, and recursive comparisons between and within incidents/concepts and theoretical coding. Finally [4], the themes were re-organized in an analytical order, enabling connections between themes, sub-themes, and codes [41]. To increase trustworthiness [42], all interviews were analyzed independently by the researcher and by a research assistant who is experienced in conducting qualitative studies. The citations were translated into English for the purpose of this article, following their double translations by the researcher and a professional English language editor who is a native English speaker.

## Results

The participants' characteristics are described in Table 1. Data analysis led to the emergence of five themes: (1) Facilitators for supporting an individual requesting suicide tourism; (2) choosing death and actively making the decision to die; (3) the meaning of traveling to die; (4) offering support throughout the process; and (5) facilitating procedures after death.

**TABLE 1:** Participant DEMOGRAPHICS (Israel, 2019–2022).

Gender (as reported)	5 females; 5 males
Age (years)	40–64 years; mean = 56 years
Marital status	5 married; 1 in a relationship; 3 widow; 1 single
Main work	4 free professions; 2 scientists; 2 managed a business; 1 employee in a large organization; 1 had a key position in an NGO.
Education	5 Ph.D.; 3 B.A.; 2 high-school education
Nationality	10 Jews
Geographical residence	4 outside Israel; 3 Central region; 2 Jerusalem region; 1 Northern region
Relationship to the deceased	7 family ties (3 the deceased’s child; 1 sibling; 1 spouse; 1 daughter-in-law; 1 remote family member); 3 other ties (2 the deceased’s close friends; 1 the deceased’s therapist
The deceased’s medical condition	5 cancer; 2 fibromyalgia and physical disabilities; 2 multi-morbidity including cognitive decline; 1 Parkinson’s disease

### Theme 1: Facilitators for Supporting an Individual Requesting Suicide Tourism

In their interviews, the participants referred to six sub-themes that enhanced their ability to support the individual in their decision to embark on suicide tourism:

(1) The individual was a very autonomous person, as seen in the following quotes: “You have to be a very special person, a very strong person, to be able to do it” (P., daughter of the deceased); and “She insisted on dealing with it alone, she never wanted to bother anyone” (T. daughter-in law of the deceased).
(2) The individual enjoyed the legitimacy of their decision. For example, “She was like, “If you can’t help cure me, then at least help redeem me from my misery”” (J., close friend of the deceased). Yet legitimizing the deceased’s decision to travel for aid-in-dying was not always easy for the family member: “I wasn’t happy with his decision to die earlier than necessary just so that he wouldn’t have to go into a hospice. At first, I didn't agree with him” (O., wife); “I told some friends that I was going abroad with my dad and I’d be coming home with a coffin. They were shocked” (P.). “I wouldn’t have done it … I thought it would be too painful for his son and grandchildren” (K., relative). Some participants spoke of the religious aspect of suicide, which is forbidden by Jewish law: “I told one of my co-workers about the plan, he’s Ultra-Orthodox. He just looked at me and said: “You know that you are a killer’” (P.).
(3) The deceased exercised control over their disease, rather than be controlled by it. For example: “She said, ‘I’m not going to become one of those poor widows who deteriorates and lives with her children. I don’t want to be a burden on them” (N., son). During the interviews, the participants spoke of the deceased having exercised control. Some made preliminary plans: “She saved newspaper cuttings. It was important for her to know that she had something to hold onto” (N.). Others placed great emphasis on the details: “In EXIT, it [the pentobarbital] is administered intravenously … She didn’t think she’d have the courage to drink it… So she knew that when the time came, she would choose EXIT” (N.). Finally, some left explicit instructions: “He left specific instructions about who would inherit what he leaves behind… and details about his bank accounts to pay for Switzerland. Everything was very specific” (L., brother).
(4) The participants also exercised a certain degree of control, to somewhat limit the individual’s plans: “I said… I won’t actively help you. If you want to organize your own death, do it yourself” (N.); and “He knew that I wouldn't let him do it. I’d give him hell just so he wouldn’t die” (L.).
(5) Participants managed differences between themselves and the individual. They spoke of their experience of dissonance between their desire to help the individual and their objections to their plans for suicide tourism. For example: “I asked him to wait. I said, “You don’t have to do this now.” I wouldn’t even take him to the doctor. I was quite manipulative” (O.); “I told her, “You’ve got 3-4 months [to live]. Let’s do things.” I was in the mode of helping her do things, talk to people, like it’s now or never. But that wasn’t what she wanted at all” (J.). When this did not work, the participants developed strategies for overcoming these gaps to support the individual: “You have to put aside your own emotions and desires and give them [the patient] priority, because this is what they want… They’ve asked you to do it and it doesn’t matter what you think or feel about it” (P.); and “The more time I spent with her, the more I became convinced by her logic. It was as if I began to see that she really wasn’t living anymore” (J.).
(6) Finally, the participants’ responses to others also facilitated their ability to support the individual: “I was very clear. I told everyone that they’re welcome to show their love, but if they are here to object [to suicide tourism], then they should go home. And if they want to cry, they should go into the kitchen” (P.).

### Theme 2: Choosing Death and Actively Making the Decision to die

In their attempt to support and accompany the individual throughout their journey, the participants grappled with the concept of death, active decision making, and the choice to die. They re-conceptualized death as “a shift towards a different energetic phase” (Q., the deceased’s therapist) and “the end of being punished through suffering and a place of peace and rest” (P., daughter of the deceased).

The participants explained that the entire process for arranging the suicide tourism takes a long time; yet this so-called downside enabled them to begin to come to terms (at least to a certain degree) with the individual’s decision. Most participants expressed complex attitudes towards their relative’s decision to die. Yet in some families, discussing death and dying was commonplace: “Death was always present. We were very cynical about it, never bothered by it” (M., son of the deceased). Others spoke of their fears about death and dying: “The sanctity of life doesn't permit us to end our lives; the thought fills us with fear. End-of-life and euthanasia include everything: psychology, morality, and religion” (L., brother). Yet most participants stated that in some circumstances, choosing to end their life could actually help the individual to continue living, possibly with less fear about their continued deteriorating or increased pain and suffering – knowing that they have a plan set in place.

The interviews also revealed that the individual’s choice to die, and the meticulous planning that is entailed in doing so, enabled the participants to facilitate, support, and even be a part of the “event,” at home before leaving for Switzerland and/or throughout the overseas journey. For example, participants mentioned they were able to truly say goodbye: “We told our friends that dad would be traveling. And that started a sort of [goodbye] period where we sat Shivah [the 7-day Jewish mourning period] while dad was alive, because people came to visit him” (P.); “She insisted that we make her last days enjoyable. And we did. We went to a chocolate factory that she remembered from her childhood, see showed us where she grew up… We wouldn’t have had these experiences if she’d been hooked up to tubes or had deteriorated.” (N.); and “We knew this was goodbye, but we also knew when and where [she was going to die]. So I got to ask her lots of questions that I probably wouldn’t have otherwise. I even asked her about the guys she’d dated… It was amazing because she felt free to answer everything I asked. She wasn’t usually the kind of person who talked about these things” (T., daughter-in law).

### Theme 3: The Meaning of Traveling to die

In this third theme, the participants expressed their concerns about the need to travel overseas to receive aid-in-dying. They spoke of the tiring bureaucratic procedures entailed in suicide tourism, and the difficulty in attaining all necessary documents: “They kept asking for more and more documents and wanted to know if we’d checked this and that” (H., a close friend of the deceased). The participants also spoke of having to communicate in English, and the need to also arrange the returning of the body from Switzerland to Israel for burial: “The paperwork took a few days to complete, until Dignitas signed the documents, and the [Israeli] Consulate signed the documents, and the company was schedule the flight back for the coffin. And in the meantime, we were trying to arrange the burial here” (P., daughter of the deceased).

The participants also complained about the financial burden of suicide tourism: “After dad died, mom sold her house to pay back the loan that she took out for this” (P.). Additionally, since the death takes place outside the country, not all family members can travel overseas to be by their relative as they take their last breath: “We closed the door behind us, leaving my mom and sister behind. I could hear them crying… It was easier for me because I was busy doing things. They only had the stress of waiting” (P.). Yet the participants also talked about their feeling capable of supporting and accompanying the individual: “I felt that I was tough enough and mature enough. I’d already had some experience with death from my military service, so I felt ready” (J., close friend).

Participants regarded suicide tourism as a phenomenon that allows people to die with dignity, rather than in a tragic or violent act, and at the same time prevents trauma to those who surround the individual at stake. Due to the legal prohibition against assisted suicide in Israel, some participants feared that they may be putting themselves at risk by participating in this practice, albeit outside the country. Still, this did not deter them from supporting their relative: “I thought to myself, “What if they want to arrest me?” One day, they might search for proof that I actually paid to have someone killed” (H.); and “We thought it could happen, but we weren’t afraid. I said to myself: I’m willing to sit in jail or anything else that will make the headlines” (N.). Yet, due to legal (and other) issues, the participants did not always reveal the truth about the event, before or after. For example, when they asked the individual’s physician in Israel for their medical records, which they needed for arranging the procedure, they only said that they need it to receive treatment overseas, without specifying the type of treatment involved. One participant asked the Swiss authorities to write “unnatural death” on the death certificate instead of “suicide,” as by Jewish law, people who commit suicide must be buried outside the regular cemetery.

Finally, the participants conveyed that public discourse on death and dying in Israel should be expanded, for normalizing and regulating the practice of aid-in-dying: “It’s common practice in Switzerland… It’s not like here. There, people know that at some point, when they get old, they can end their life” (N.).

### Theme 4: Offering Support Throughout the Process

Most participants provided support after having specifically been chosen by the individual. This was based on their relationship between the two, or in light of certain characteristics or beliefs among the participants, for example not being critical by nature, or having no financial interest in the individual’s death. The participants justified their offering support through general concepts, such as “all humans should be surrounded by love in these situations” (N.); “It’s the most meaningful human gesture: (M., son of the deceased); “I find it hard to believe that a person who wants to die will do so alone. You need strength, focus, and bureaucratic abilities and if you’re in pain or on medication, you just can’t do it” (H.).

Some participants spoke of the specific actions that they took to support the individuals on their suicide-tourism journey, including assisting them with paperwork and communications with Dignitas, accompanying them to Switzerland, providing them with emotional support, arranging the disposal of the body and or funeral, and communicating with friends and family following the event. They also spoke of being present during the death: “I was afraid to go there. To watch how they do it.” (O.); “The role division was very clear. They drive here there, accompany her, but I am the manager of this case.” (H’). Whereas for others, being present at the moment of death was perceived as highly meaningful and a very brave act of support (“I held the glass for her… I was waiting to see her hesitate, but nothing! She was very goal oriented… and within 30 s, she wasn’t with us anymore” (M.).

Finally, some participants spoke of their own need for support during this process, from the moment they were informed about the individual’s wishes, and even more so throughout the process. They referred to seeking informal means of support, including anonymous calls to a television consulting program, conversations with other family members, friends, and even co-workers. Most stated that a more structured and organized support system is required.

### Theme 5: Facilitating Procedures After Death

In most cases, the participants held funerals and sat *Shivah* following the death, which was especially meaningful given that the individual had travelled overseas to die, usually unbeknown to most of the deceased’s family members and friends. The *Shivah* enabled closure for many people, including those who opposed the individual’s plan to die.

Upon returning to Israel, the participants experienced various reactions to the individual’s death, the manner in which it occurred, and their own involvement. Some reactions were positive: “Some said Bravo, well done to her!” (N.). Others were more critical: “There was one guy who just couldn’t understand it. He said, “How did you do it? How did you kill your mother? How did you go with her?”” (M., son); and “During the Shivah, people started expressing their opinions, like “He wasn’t that sick. He didn’t have to travel … ”” (P., daughter of the deceased).

## Discussion

This study explores the experiences, attitudes, and challenges faced by people who supported a family member or close friend on their journey to end their life and suffering in Switzerland. The findings of this study reinforce the literature whereby those who are actively engaged in end-of-life through assisted suicide are motivated and determined individuals who believe that it is their moral and legal right to put an end to their suffering, pain, and dependency [17, 19]. The findings also highlight the paradox whereby individuals who wish to end their life so as not to become a burden on others actually place a different type of burden on the relative who they ask to support them throughout this journey [43].

Despite assisted suicide being forbidden in Israel, and little discourse and media coverage on the topic [44], some Israelis choose to embark on suicide tourism [45], as seen in this study. While they have a strong desire to end their lives, based on wish to die and the ideal of an aesthetic death, that could be secured in such a journey [22, 23], they still seek a certain degree of legitimacy and acceptance from someone close. The participants’ involvement throughout the process confirms previous claims whereby relationships are central to end-of-life processes and decision-making [27, 46, 47]. This may be even more so in the case of suicide tourism, where the individual and their “supporter” must travel overseas.

The participants also spoke of their active role in the process, even when experiencing conflicting emotions and values regarding the decision, as seen in previous studies [27, 48]. Yet unlike [49], who found that family caregivers describe their experience as participating in a “race to the end,” while attempting to create an ideal dying experience for the patient, some participants in this study questioned the individual’s decision, even actively postponing the execution of their plan or setting certain limitations. These findings therefore add to the literature, whereby family members take responsibility regarding their loved-ones’ wish to die, implementing their subjective moral obligation to actively prevent or assist them in carrying out their wishes [50].

By providing support for the individual seeking suicide tourism, the participants in this study were also able to exercise control, allowing time for others to say goodbye, for example, and reducing their suffering and burden. In their systematic review of the literature, Singer et al. [51] discuss both protective and risk factors regarding the bereavement outcomes of family members, following the medical aid-in-dying of a loved one. Indeed, in the current study, the participants expressed their need for support for themselves throughout the process, as they often felt alone, overwhelmed, or conflicted. Pronk et al. (2023) also found that not only are such deaths painful for those who are left behind, but they could even complicate the latter’s grieving process. As such, establishing support groups, ethical consultations, and professional guidance for relatives in such situations—combined with training on assisted suicide bereavement for professionals—could be beneficial for dealing with aid-in-dying processes and deaths [33, 48, 52].

Interestingly, in a previous study [36], family members spoke of the actual journey overseas and the challenges that this entailed; in the current study, however, the participants rarely spoke of specific characteristics of their travel experiences, expanding instead on the more symbolic and meaningful aspects related to dying overseas. It may be that in this study, the individuals at stake did not suffer from physical limitations impairing their ability to travel or that such a travel had not been experienced by participants as too demanding. Moreover, contrary to studies where family members spoke of their isolation and difficulties in sharing their story after the death, fearing people’s possible reactions and social stigmas [29, 53], the participants in this study reported being ready and willing to deal with the reactions of others regarding their involvement in the deceased’s plan. It is likely that the Jewish ritual of the 7-day *Shivah,* in which visitors continuously come to the house of the deceased to offer their condolences and support for the remaining family members [54] serves as a positive informal means for the participants to process their role in the event.

### Limitations

This novel study contributes to the literature on aid-in-dying, with an emphasis on the relative who offered support during the process. Yet a number of research limitations should be addressed. First, all interviews were conducted by the researcher; yet the data, which was accurately recorded and transcribed, were also analyzed by an additional researcher, along with the author ensuring reflexivity during the analysis. Moreover, a relatively small number of participants took part in this study. However, with qualitative research, the sample size is determined by the context and the information the sample holds [55]. Moreover, in this case, due to ethical and legal reasons, suicide tourism in Israel is not widespread, and identifying those who did participate in such events is extremely difficult. As such, the current sample size could be considered adequate. Additionally and regardless of sample size, the participants' narratives and attitudes conveyed during the interviews enabled an in-depth understanding of their subjective experiences and meanings of the phenomenon [56].

### Conclusion

This study explored the personal experiences, perspectives, and attitudes of family members and friends who supported individuals who chose to end their lives through suicide tourism, traveling from Israel to Switzerland to do so. The findings highlight their facilitating role in supporting these individuals before and after the assisted suicide, especially in light of the need for ongoing bureaucratical processes and traveling to another country; they also relayed their own experiences and emotions regarding death by choice. This study provides important implications for services related to end-of-life planning and for reshaping related societal values.
