# Supplementary material for: Views, Attitudes and Challenges When Supporting a Family Member in Their Decision to Travel to Switzerland to Receive Aid-In-Dying
Source: Int J Public Health. 2024 Jun 24;69:1607410. doi: 10.3389/ijph.2024.1607410 (PMC11228154; doi:10.3389/ijph.2024.1607410)
Supplement: Supplementary file 1 [file DataSheet1.docx]

**Annex 1: Interview guide**

1. Please introduce yourself, your age, education, and work experience.
2. Please tell me about your relationship with the deceased (X).
3. Please tell me about the disease of X.
4. How well did X function on a daily basis?
5. How did X regard his/her condition?
6. How did the deceased hear about Dignitas?
7. What did you think about X's intention to end his/her life?
8. How did X's decision make you feel?
9. What were the deceased's expectations from you concerning his/her plan?
10. What preparations did you make for this plan?
11. Did you accompany X to Switzerland? Please tell me what thoughts you had on this.
12. Which challenges did you encounter while being involved in X's plan?
13. Please describe the very last moments before X died.
14. How did you feel after the deceased died?
15. What actions did you take following X's death?
16. What were you concerned about when you returned home / after the death?
17. Looking back, how would you regard your involvement and support of the deceased in the journey to end his/her life in Switzerland?
18. Do you have any other issues that you would like to raise?
